# Zein–Curcumin Composite Edible Films for Intelligent Packaging: A Natural pH-Sensing Indicator to Monitor Sea Bream Freshness

**DOI:** 10.3390/foods14223846

**Published:** 2025-11-10

**Authors:** Burcu Demirtas, Beyza Keser, Serpil Tural, Latife Betül Gül, Ilay Yilmaz, Mahmut Ekrem Parlak, Ayşe Neslihan Dündar, Maria D’Elia, Luca Rastrelli, Furkan Turker Saricaoglu

**Affiliations:** 1 Department of Food Engineering, Faculty of Engineering and Natural Science, Bursa Technical University, 16310 Bursa, Türkiye; burcudemirtas.bd50@gmail.com (B.D.); beyzaksr1@gmail.com (B.K.); ilayyilmaz1@hotmail.com (I.Y.); ekrem.parlak@btu.edu.tr (M.E.P.); ayse.dundar@btu.edu.tr (A.N.D.); 2 Samsun Food Control Laboratory Directorate, Ministry of Agriculture and Forestry, 55200 Samsun, Türkiye; serpil.tural@tarimorman.gov.tr; 3 Department of Food Engineering, Engineering Faculty, Ondokuz Mayıs University, 55200 Samsun, Türkiye; latifebetul.gul@omu.edu.tr; 4 Department of Pharmacy, University of Salerno, 84084 Salerno, Italy; mdelia@unisa.it; 5 National Biodiversity Future Center (NBFC), 90133 Palermo, Italy; 6 Dipartimento di Scienze della Terra e del Mare, University of Palermo, 90128 Palermo, Italy

**Keywords:** curcumin, zein, edible films, pH indicator, intelligent packaging, fish freshness

## Abstract

This study developed and characterized zein-based edible films enriched with curcumin as natural pH-sensitive indicators for monitoring fish freshness. Colorimetric films were prepared with different curcumin concentrations (1–7% wt) and evaluated for physicochemical, mechanical, optical, and antioxidant properties. Increasing curcumin content reduced water vapor permeability (0.085–0.110 g·mm/m^2^·h·kPa), lowered water contact angles (<90°), and enhanced hydrophilicity. Films exhibited high brightness, with decreased a* and increased b* values, while light transmission decreased, improving UV barrier properties. Colorimetric response (ΔE*) across pH 3–10 was more pronounced at higher curcumin levels, confirming pH-sensitivity. Antioxidant activity significantly increased with curcumin loading (up to 24.18 µmol Trolox/g). Mechanical analysis revealed decreased tensile strength but improved elongation at break, bursting strength, and deformation, supported by SEM images showing more homogeneous, micro-porous structures at 7% curcumin. Zein films containing 7% (wt) curcumin (Z/CR7) were applied to gilthead sea bream (*Sparus aurata*) fillets stored at 4 °C for 13 days. Results showed lower TBARS and TVB-N values in Z/CR7 compared to the control, indicating delayed lipid oxidation and spoilage. Colorimetric changes in the films corresponded with fish freshness deterioration, providing a clear visual indicator. Microbiological results supported chemical findings, though antimicrobial effects were limited. Curcumin-enriched zein films demonstrated strong potential as intelligent, biodegradable packaging for real-time monitoring of seafood quality.

## 1. Introduction

The growing need to replace petroleum-based plastics has stimulated the development of biodegradable packaging derived from agricultural by-products. Among these, edible films are attractive because they are safe, renewable, and capable of providing protection against physical, chemical, and microbiological deterioration [1,2]. When bioactive compounds are incorporated, such films can evolve into intelligent packaging systems, acting not only as barriers but also as freshness indicators through optical or functional responses [3].

Proteins are particularly suitable as film-forming agents. Zein, the alcohol-soluble prolamin of corn, is widely available as a by-product of corn and bioethanol industries. It displays film-forming ability, hydrophobicity, and biodegradability, though its mechanical and barrier performance can be further improved by blending or introducing functional additives [4,5]. A growing strategy is the incorporation of natural pigments into zein films, conferring colorimetric sensitivity and expanding their application in intelligent packaging. Recent reviews highlight that zein-based films have attracted great interest as biodegradable materials due to their high transparency, oxygen barrier properties, and moisture resistance, but they require modification with plasticizers, nanoparticles, polyphenols, or other biopolymers to overcome brittleness and enhance flexibility. Importantly, zein’s role in intelligent response systems, such as pH- or freshness-indicating films, has been recognized as a promising development direction for food preservation [6].

Curcumin, the principal polyphenolic compound in turmeric (*Curcuma longa*), is well known for its strong antioxidant, antimicrobial, and anti-inflammatory activities [7,8]. Its remarkable pH sensitivity, yellow under acidic/neutral conditions and reddish-brown under alkaline ones, makes it a promising natural alternative to synthetic pH indicators, which pose potential safety risks [9]. Curcumin-based films have been successfully tested in starch and pectin matrices to indicate the freshness of perishable products such as shrimp and fish. For instance, Chen et al. (2020) [10] demonstrated effective spoilage detection in fish, although the starch/PVA films exhibited limited mechanical strength. Ezati and Rhim (2020) [11] extended the approach to starch–pectin matrices, achieving improved antioxidant activity but reporting high water solubility, which could compromise film stability. More recently, Mohseni-Shahri and Moeinpour (2023) [12] showed that combining curcumin with *Hibiscus sabdariffa* anthocyanins in gelatin films enhanced pH sensitivity during shrimp storage, although the use of animal-derived gelatin may restrict applications in plant-based packaging systems. Despite these advances, the direct combination of zein and curcumin, and its validation in real seafood systems, remains largely unexplored. The hydrophobic structure of zein and the insolubility of curcumin in water make these two components a promising alternative for producing pH-sensing packaging materials for highly water-content foods. Seafood represents a strategic application model for intelligent films because of its high perishability and economic relevance. Gilthead sea bream (*Sparus aurata*) is a key species in Mediterranean aquaculture, valued for its high-quality protein, polyunsaturated fatty acids, and micronutrients. However, like most seafood, it is highly perishable, with rapid spoilage occurring even under refrigeration. The short shelf life is primarily due to microbial proliferation, accumulation of volatile amines, and lipid oxidation, which collectively compromise sensory quality and safety [13,14,15]. For these reasons, seafood products are often regarded as sensitive food models for evaluating the efficacy of intelligent packaging systems. Traditional monitoring relies on destructive and time-consuming chemical or microbiological analyses, whereas intelligent colorimetric films can provide a rapid, non-invasive freshness indicator directly visible to consumers and industry.

Therefore, this study aimed to develop zein-based edible films incorporated with curcumin as a natural pH-sensitive compound, and to characterize their physicochemical, mechanical, and functional properties. Furthermore, the applicability of the optimized film was evaluated in monitoring the freshness of gilthead sea bream during refrigerated storage. The outcomes of this research provide insights into the development of sustainable, intelligent packaging systems capable of enhancing food quality control and reducing waste in seafood supply chains.

## 2. Materials and Methods

### 2.1. Materials

Turmeric (*Curcuma longa*) rhizome powder and fresh sea bream (*Sparus aurata*; 47.54 ± 4.92% dry matter, 40.09 ± 1.40% protein, 18.70 ± 1.40% fat, 0.82 ± 0.15% ash) were purchased from local suppliers in Bursa, Türkiye. Sea bream was transported to the laboratory in ice within 30 min, packed in sealed bags, and stored at −20 °C until use. All chemicals used were of analytical grade. Food-grade corn zein (protein content 98%) from *Zea mays* L. was purchased from J&K Scientific LLC (San Jose, CA, USA). Polyethylene glycol (PEG-400) was used as a plasticizer, and ethanol (96% *v*/*v*) was obtained from Merck (Darmstadt, Germany).

### 2.2. Preparation of Curcumin Extract

Curcumin (CR) was extracted from turmeric rhizome powder using 70% ethanol at a ratio of 1:9 (*w*/*v*) under stirring (Daihan MSH—20D, Seoul, Republic of Korea) at 40 °C for 1 h in the dark. The extract was refrigerated for 24 h and then filtered through filter paper. The filtrate was dried in an air-circulated oven (Nukleon Lab, Ankara, Türkiye) at 60 °C overnight. The dried curcumin was stored in sterile containers at −18 °C until further use.

### 2.3. Preparation of Films

Zein-based films were prepared by dissolving 6.75 g of zein in 100 mL of an ethanol:water mixture (95:5, *v*/*v*) under magnetic stirring at 70 °C for 30 min. PEG-400 was added as a plasticizer at 30% (*w*/*w*) of zein, and the solution was centrifuged at 7000 rpm for 15 min to remove undissolved debris and air bubbles. The clear film-forming solution was cast onto glass plates coated with Mylar film and spread using a roller to achieve a wet thickness of 1.143 mm. Films were dried at room temperature (~24 h), peeled from the plates, and stored in a light-proof cabinet between filter papers.

**Figure 1:**
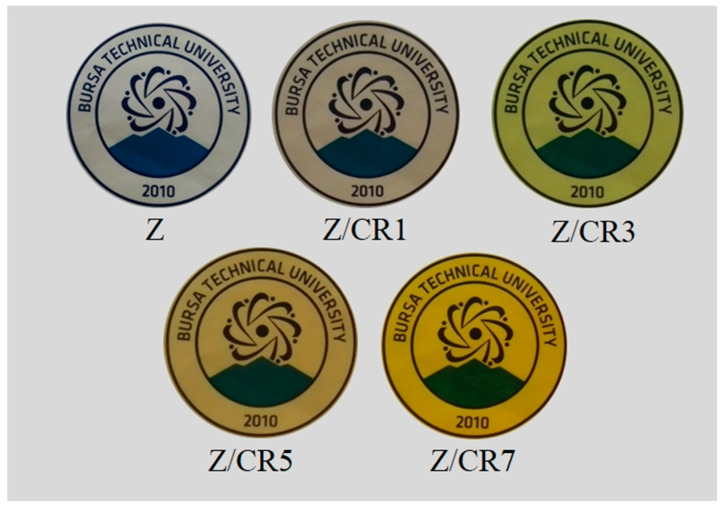
Visual appearance of zein films enriched with curcumin at different concentrations. Z: Neat zein film; Z/CR1: Zein film containing 1% curcumin; Z/CR3: Zein film containing 3% curcumin; Z/CR5: Zein film containing 5% curcumin; Z/CR7: Zein film containing 7% curcumin.

For curcumin-enriched films, curcumin was incorporated into the zein solution at 1%, 3%, 5%, and 7% (*w*/*w*, based on polymer mass) and mixed for an additional 30 min before casting (Figure 1). All other steps were identical to the control films. Before analysis, film samples were conditioned for 3 days at 53% relative humidity in a desiccator containing a saturated magnesium nitrate (Mg(NO_3_)_2_) solution.

### 2.4. Characterization of Curcumin

#### 2.4.1. DPPH• Free Radical Scavenging Activity

The antioxidant capacity of curcumin was determined using the 2,2-diphenyl-1-picrylhydrazyl (DPPH•) assay with slight modifications. Briefly, 250 mg of curcumin was dissolved in 5 mL of 70% ethanol. An aliquot of 0.1 mL of this solution was mixed with 5.9 mL of DPPH• solution. After vigorous shaking, the mixtures were incubated in the dark at room temperature for 30 min. Absorbance was measured at 517 nm using a UV-Vis spectrophotometer (Rigol Ultra 3660, IndiaMart, Noida, India). Antioxidant capacity was calculated from a Trolox calibration curve and expressed as µmol Trolox equivalents per gram of curcumin (µmol Trolox/g).

#### 2.4.2. Spectral Characteristics of Curcumin

To evaluate the pH-dependent spectral properties, 500 mg of curcumin was dissolved in 10 mL of 70% ethanol. One milliliter of the solution was then mixed with 3 mL of buffer solutions at pH values ranging from 3 to 10. UV—Vis spectra were recorded over the wavelength range of 200–800 nm.

### 2.5. Characterization of Zein-Based Films

#### 2.5.1. Thickness, Moisture Content, Swelling Index, and Solubility

Film thickness was measured using a digital micrometer (293–821 model, Mitutoyo, Kawasaki, Japan) with a sensitivity of 0.001 mm at 16 random points. Film samples (2 × 2 cm) were dried at 105 °C for 24 h, and the moisture content (MC) was calculated from the weight loss. For swelling index (*SI*), dried specimens were weighed, immersed in 30 mL of distilled water, and stored in a climate chamber at 40–45% relative humidity for 24 h. Excess surface water was gently removed with filter paper before reweighing. *SI* was calculated using Equation (1):

(1) $$SI\;(\%)=\frac{W_{f}-W_{i}}{W_{i}}\times 100$$

where $W_{f}$ and $W_{i}$ are the weights of swollen and dry films (g), respectively.

Water solubility was determined by drying the residual solution at 105 °C for 24 h. The weight difference between the empty Petri dish and the final dried dish was used to calculate solubility (%).

#### 2.5.2. Water Vapor Permeability (WVP)

Water vapor permeability was determined using the modified gravimetric cup method in accordance with ASTM E96–80 [16]. Circular film specimens (6 cm diameter) were prepared, and the thickness was measured at five points. Films were sealed onto PMMA (poly(methyl methacrylate)) cells (opening diameter 50 mm) containing 6 mL of distilled water. The cells were placed in a controlled-humidity cabinet (0% RH) containing anhydrous silica. Weight loss was recorded every 2 h for 48 h using an analytical balance. Each treatment was analyzed in triplicate. *WVP* was calculated using Equation (2):

(2) $$WVP\;=\frac{W}{t}\times \frac{x}{∆P\times A}$$

where *W*/*t* is the weight change over time (g/h), *x* is film thickness (mm), Δ*P* is the water vapor pressure difference (kPa), and *A* is the exposed film surface area (m^2^).

#### 2.5.3. Water Contact Angle

The surface hydrophobicity of zein films containing different concentrations of curcumin was evaluated using the sessile-drop method with a contact angle goniometer (Attension Theta, Biolin Scientific, Vastra Frolunda, Sweden). A 5 μL drop of distilled water was placed on the film surface, and the contact angle between the drop and the surface was immediately measured. Images of the droplets on the film surface were recorded for analysis.

#### 2.5.4. Optical Properties

Color parameters (L*, a*, and b*) of the films were measured at five random points on the film surface using a colorimeter (HunterLab UltraScan VIS, Reston, VA, USA). Light transmission was determined in the range of 200–800 nm at 10 nm intervals with a UV—Vis spectrophotometer (Rigol Ultra 3660, India). Film samples were cut into rectangular strips (4 × 1 cm) and placed in quartz cells for measurement. Film opacity was determined according to Kurt and Kahyaoglu (2014) [17]. Absorbance at 600 nm was recorded, and opacity was calculated as absorbance divided by film thickness.

The pH sensitivity of the films was assessed by immersing curcumin-enriched zein films (cut into 4 × 4 cm pieces) in buffer solutions with pH values ranging from 3 to 10 for 30 min. The resulting color differences (ΔE*) were calculated relative to control zein films without curcumin, using Equation (3):

(3) $$∆E=\sqrt[{2}]{\left({\left(L_{0}^{*}-L_{1}^{*}\right)}^{2}+{\left(a_{0}^{*}-a_{1}^{*}\right)}^{2}+{\left(b_{0}^{*}-b_{1}^{*}\right)}^{2}\right)}$$

where $L_{0}^{*}$, $a_{0}^{*}$, and $b_{0}^{*}$ are the color values of the control film, and $L_{1}^{*}$, $a_{1}^{*}$, and $b_{1}^{*}$ are the corresponding values of the curcumin-enriched films after immersion in buffer solutions.

#### 2.5.5. Mechanical Properties

Tensile strength (TS) and elongation at break (EAB) of the films were determined using a texture analyzer (TA—HD Plus, Stable Micro Systems, Godalming, UK) according to ASTM D882 [18] with minor modifications. Film strips (1 × 8 cm) were conditioned at 54% relative humidity (RH) for 3 days, mounted on the tensile grips with an initial gap of 6 cm, and stretched at a crosshead speed of 2 mm/s until fracture. Force and extension were continuously recorded. At least eight replicates were analyzed for each film formulation.

Burst strength (BS) and burst distance (BD) were measured on 3 × 3 cm film samples using a film support ring (10 mm diameter, HDP/FSR platform) fitted with a spherical probe (SMS P/0.25S, 6.25 mm diameter). The probe moved at 1 mm/s until the film ruptured, and both the maximum rupture force and deformation distance were recorded. Each treatment was tested in triplicate.

#### 2.5.6. Thermal Properties

Thermal properties of the films were evaluated using differential scanning calorimetry (DSC) (Discovery 251, TA Instruments, New Castle, DE, USA). Before analysis, films were conditioned in a desiccator with anhydrous silica for 3 weeks to remove residual moisture. Samples (4–5 mg) were heated from 0 to 250 °C at a rate of 10 °C/min under a nitrogen flow of 20 mL/min.

#### 2.5.7. Fourier Transform Infrared Spectroscopy (FTIR)

The chemical interactions between zein and curcumin were investigated using attenuated total reflectance–Fourier transform infrared (ATR—FTIR) spectroscopy (Bruker Alpha II, Manheim, Germany). Spectra were collected in the range of 450–4500 cm^−1^ with a resolution of 4 cm^−1^, averaging 12 scans per sample. The infrared spectrum of pure curcumin was also recorded for comparison. Before analysis, all film samples were conditioned in a desiccator containing silica gel for 10 days to minimize moisture interference.

#### 2.5.8. Scanning Electron Microscopy (SEM)

The surface and cross-sectional morphologies of the films were examined using a scanning electron microscope (Carl Zeiss Gemini 300, ZEISS, Jena, Germany). Samples were mounted and sputter-coated with a thin layer of gold–palladium in a vacuum chamber (Leica Microsystems, EM ACE600, Wetzlar, Germany) to enhance conductivity. Images were acquired at magnifications of 1000× (surface) and 750× (cross-sections).

### 2.6. Application of Films for Monitoring Real-Time Freshness of Fish

Sea bream fillets (100 g each) were individually packaged in polyethylene terephthalate (PET) trays and stored at 4 °C for up to 16 days. Zein films containing 0% curcumin (Z) and 7% curcumin (Z/CR7) were attached to the inner surface of the tray, without direct contact with the fish. A control group without film (CNT) was also included. Fish quality was evaluated on storage days 0, 3, 8, and 13. Each parameter was analyzed in duplicate, and the results are expressed as mean values.

#### 2.6.1. pH

The pH of fish samples was determined using a digital pH meter (Ohaus ST3100, Ohaus, Parsippany, NJ, USA). Measurements were taken from three different points of each sample. For analysis, 10 g of fish was homogenized in 90 mL of distilled water (IKA T25 homogenizer, Staufen, Germany) for 1 min, and pH was recorded directly from the homogenate.

#### 2.6.2. Thiobarbituric Acid Reactive Substances (TBARS)

Lipid oxidation was assessed by measuring thiobarbituric acid reactive substances (TBARS) as previously reported [13]. Briefly, 10 g of fish was homogenized with 20 mL of distilled water and 25 mL of 20% trichloroacetic acid (TCA) for 2 min (IKA T25 homogenizer). The homogenate was filtered through Whatman No. 1 filter paper, and 5 mL of the filtrate was mixed with 5 mL of 0.02 mol/L thiobarbituric acid (TBA) reagent. The mixture was heated in boiling water for 35 min, rapidly cooled, and the absorbance was measured at 532 nm against a reagent blank. A calibration curve was prepared with 1,1,3,3-tetraethoxypropane, and TBARS results were expressed as mg malondialdehyde (MDA)/kg of sample.

#### 2.6.3. Total Volatile Basic Nitrogen (TVB—N)

TVB—N content was determined using the Kjeldahl distillation method. Fish fillets (5 g) were homogenized with 45 mL of 0.6 M perchloric acid using an Ultra-Turrax homogenizer (Velp Scientific, OV-5, Alpnach, Switzerland) at 5000 rpm for 5 min. The homogenate was centrifuged (Nüve 3000R, Ankara, Türkiye) at 3000 rpm for 10 min at 25 °C, and the supernatant was filtered through Whatman No. 1 filter paper. The filtrate was transferred to a Kjeldahl distillation unit (Buchi B-324, Buchi, Flawil, Switzerland), mixed with 25 mL of 30% NaOH, 3 g MgO, and 1.5 mL defoamer, and distilled. The distillate was collected into a conical flask containing 50 mL of 40 g/L boric acid with phenolphthalein as indicator and titrated with 0.01 M HCl. *TVB—N* values were expressed as mg N/100 g of sample, calculated using Equation (4):

(4) $$TVB-N=\frac{\left(V_{t}-V_{b}\right)\times c\times 14}{m}\times 100$$

where *V_t_* and *V_b_* are the titration volumes of sample and blank (mL), *c* is the HCl concentration (M), and *m* is the sample weight (g).

#### 2.6.4. Color Changes of Films and Fish Fillets During Storage

Color parameters (L*, a*, b*) of the films attached to the package headspace and of fish fillets were recorded on storage days 0, 3, 8, and 13 using a colorimeter (HunterLab UltraScan VIS). The peeled film samples from the headspace of the packaging were used directly for color measurements, whereas the color of the fish fillets was determined from their surface.

#### 2.6.5. Microbiological Analysis

For microbial analysis, 10 g of fish fillet was aseptically transferred into a sterile stomacher bag with 90 mL of sterile saline solution (0.85% NaCl) and homogenized for 1 min to obtain the initial dilution. Serial decimal dilutions were prepared in saline solution, and appropriate dilutions were plated for microbial enumeration. Total viable count (TVC) and psychrotrophic count (PTC) were determined on plate count agar (Merck) after incubation for 5 days at 25 °C and 10 days at 7 °C, respectively. Yeasts and molds were enumerated on Dichloran Rose Bengal Chloramphenicol Agar (DRBC) after incubation for 5 days at 25 °C. Results were expressed as log CFU/g. All analyses were performed in triplicate.

### 2.7. Statistical Analysis

Film preparation was performed in triplicate, and all characterization analyses were conducted in duplicate, except for microstructural imaging, which was carried out once. Data were expressed as mean ± standard deviation. Statistical analysis was performed using one-way analysis of variance (ANOVA), and mean differences were compared using Duncan’s multiple range test at a significance level of *p* < 0.05 (SPSS v22.0, IBM Corp., Armonk, NY, USA).

## 3. Results and Discussion

**Figure 2:**
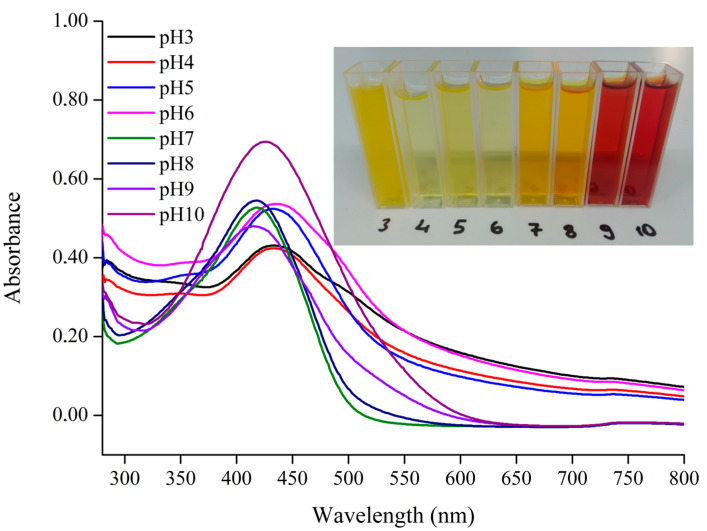
UV—Vis spectra and photographs of curcumin solutions at different pH values (3–10).

Curcumin was successfully extracted from turmeric powder using 70% ethanol, dried under vacuum, and obtained with a yield of 10.16%. The antioxidant capacity, evaluated by the DPPH radical scavenging assay, was 281.09 ± 2.73 µmol Trolox/g dry weight. This strong activity is attributed to the phenolic hydroxyl groups of curcumin, which can donate hydrogen atoms to form phenoxy radicals and neutralize free radicals [19]. The pH responsiveness of curcumin solutions was evaluated to validate their suitability as natural pH indicator dyes. As shown in Figure 2, the color of curcumin changed progressively from yellow to red when the pH increased from 3.0 to 10.0. Specifically, curcumin solutions appeared yellow at pH 3, light yellow between pH 4–6, orange at pH 7–8, and reddish-brown at pH 9–10. At pH 3.0, the solution appeared more turbid compared to pH 4.0, whereas a clear shift from yellow to orange occurred between acidic and near-neutral conditions (pH 4.0–7.0). At higher alkalinity, the solution gradually turned dark red. This phenomenon is consistent with the molecular structure of curcumin, which exhibits pH-dependent tautomerism. The keto form predominates in acidic and neutral conditions, while the enol form dominates under alkaline pH due to deprotonation of phenolic hydroxyl groups and the formation of phenoxy anions [20]. Such reversible structural transformations are responsible for the distinct color changes observed [10].

### 3.1. Characterization of Zein-Based Films

#### 3.1.1. Thickness, Moisture Content, Swelling Index, and Solubility

The thickness, moisture content (MC), water uptake (WU), and solubility of zein-based films with different curcumin concentrations are summarized in Table 1. Film thickness values ranged from 0.032 to 0.034 mm. Incorporation of 1% and 3% curcumin had no significant effect on film thickness, while films with 5% and 7% curcumin exhibited a slight but significant increase (*p* < 0.05), implying that higher curcumin concentrations modify the film-forming network. The uniformity of thickness across formulations suggests the reproducibility of the casting method. MC values decreased slightly with increasing curcumin incorporation, from 6.13% in pure zein films to 5.11% in Z/CR7 samples, although differences were not statistically significant (*p* > 0.05). This reduction may be linked to the higher total solids content and hydrophobic nature of curcumin, which can limit water retention in the film matrix. Swelling index values increased markedly with curcumin concentration (*p* < 0.05). The lowest SI was observed in the pure zein film (26.87%), while Z/CR7 films reached 76.28%. Despite curcumin being generally considered hydrophobic, the presence of hydroxyl groups in its molecular structure enables hydrogen bonding with water molecules, thereby promoting higher water absorption when incorporated into zein films [21]. Film solubility followed a similar pattern, increasing from 20.18% in pure zein films to 24.99% in Z/CR7 films (*p* < 0.05). This increase may be associated with the disruption of zein–zein interactions and the formation of additional hydrogen bonds with curcumin, which facilitated greater water penetration and dissolution of the film matrix. Comparable results were reported for zein films modified with volatile oils, where hydrophilic interactions enhanced swelling and solubility [22].

#### 3.1.2. Water Vapor Permeability (WVP)

The water vapor permeability (WVP) values of zein films with different curcumin contents are also presented in Table 1. Pure zein films (Z) exhibited the highest WVP (0.110 g·mm/m^2^·h·kPa), whereas the incorporation of curcumin progressively reduced permeability, reaching the lowest value in Z/CR7 films (0.085 g·mm/m^2^·h·kPa) (*p* < 0.05) (Table 1). This trend indicates that curcumin incorporation improved the barrier properties of zein films against water vapor. The reduction in WVP with increasing curcumin concentration may be attributed to the hydrophobic nature of curcumin molecules, which could fill voids in the polymer network and hinder water vapor diffusion through the film matrix. Moreover, possible interactions between zein and curcumin via hydrogen bonding may have led to a denser and less permeable polymer structure. Similar results were observed in zein-based films enriched with natural antioxidants and essential oils, where hydrophobic bioactive compounds reduced water vapor transmission [22]. These findings highlight that curcumin not only imparts pH-sensitivity and bioactivity but also contributes positively to the functional barrier performance of zein films, making them suitable for food packaging applications where moisture control is critical.

#### 3.1.3. Water Contact Angle (WCA)

**Figure 3:**
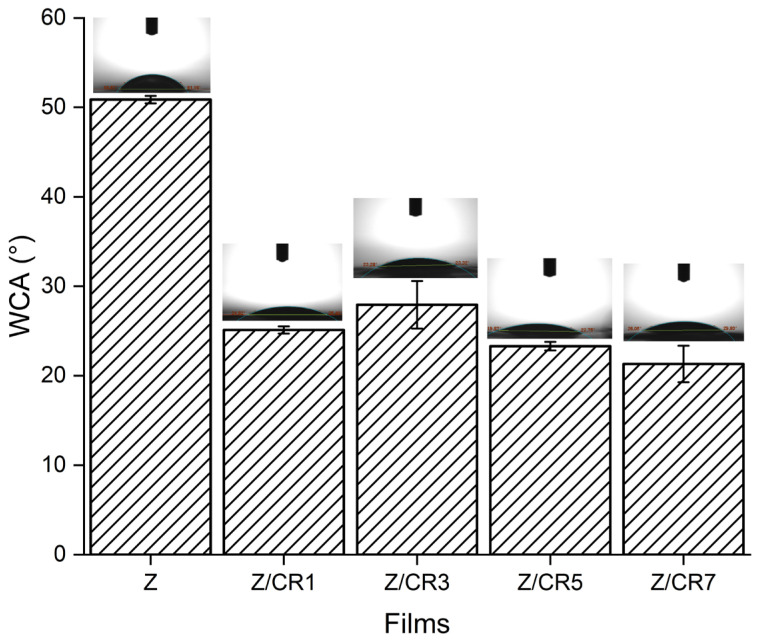
Water contact angle (WCA) of zein films containing different concentrations of curcumin (Z, Z/CR1, Z/CR3, Z/CR5, Z/CR7). Insets show representative droplet images on the film surfaces.

The surface wettability of zein films containing different amounts of curcumin was evaluated by water contact angle (WCA) measurements (Figure 3). Pure zein films (Z) exhibited the highest WCA value (≈52°), indicating their relatively hydrophobic nature. However, the incorporation of curcumin significantly decreased the WCA values (*p* < 0.05), with Z/CR7 films showing the lowest value (≈21°).

This progressive reduction in WCA demonstrates that increasing curcumin content enhanced the hydrophilicity of zein films. Although curcumin is generally described as hydrophobic, its hydroxyl and methoxy groups can form hydrogen bonds with water molecules, thereby increasing surface wettability. Furthermore, the uniform dispersion of curcumin within the zein matrix may have contributed to exposing more polar functional groups to the film surface.

Similar results have been reported for zein-based films incorporated with polyphenolic compounds, where the addition of hydroxyl-rich molecules reduced the contact angle and increased hydrophilicity [21]. Improved surface wettability may be advantageous in active packaging systems, as it facilitates interactions with aqueous food environments and can influence the release of bioactive compounds.

#### 3.1.4. Optical Properties

The visual and optical characteristics of packaging films are critical parameters that directly affect consumer acceptance. The optical properties of zein-based films enriched with different concentrations of curcumin are summarized in Table 2. The addition of curcumin significantly influenced the L*, a*, and b* values of films (*p* < 0.05). All films displayed high brightness (L* > 90), with Z/CR3 showing the highest lightness (94.41) and Z/CR5 the lowest (92.46). Zein films generally exhibited a yellowish hue, which was intensified by curcumin incorporation. Increasing curcumin concentration decreased a* values (indicating a shift towards green) and increased b* values, reflecting greater yellowness. These results are consistent with the intrinsic yellow color of curcumin and the visual appearance of the films (Figure 1).

**Figure 4:**
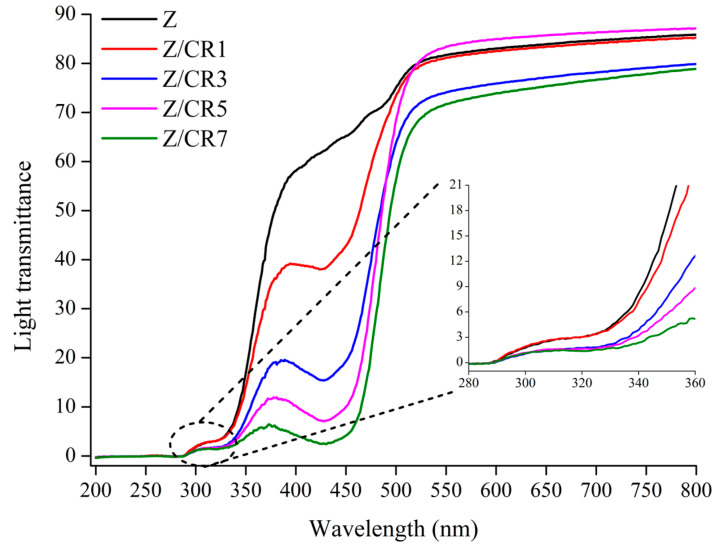
Effect of curcumin incorporation at different concentrations on light transmittance of zein-based films. Z: Neat zein film; Z/CR1: Zein film containing 1% curcumin; Z/CR3: Zein film containing 3% curcumin; Z/CR5: Zein film containing 5% curcumin; Z/CR7: Zein film containing 7% curcumin.

Light protection is another essential property of packaging films, particularly for foods rich in lipids, since UV and visible light can accelerate lipid oxidation. As shown in Table 2, the incorporation of curcumin increased film opacity, with Z/CR3, Z/CR5, and Z/CR7 showing significantly higher values than control and Z/CR1 films (*p* < 0.05). This can be attributed to the chromophoric structure and conjugated double bonds of curcumin, which imparted a yellowish tint and enhanced light absorption [23]. The reduced light transmittance, illustrated in Figure 4, demonstrates that curcumin-enriched films effectively blocked UV—B light (280–315 nm) and decreased overall light penetration, suggesting their potential to protect light-sensitive foods. Comparable findings were reported in pectin-based films enriched with curcumin, where reduced light transmittance was linked to the strong absorption of curcumin [24,25].

**Figure 5:**
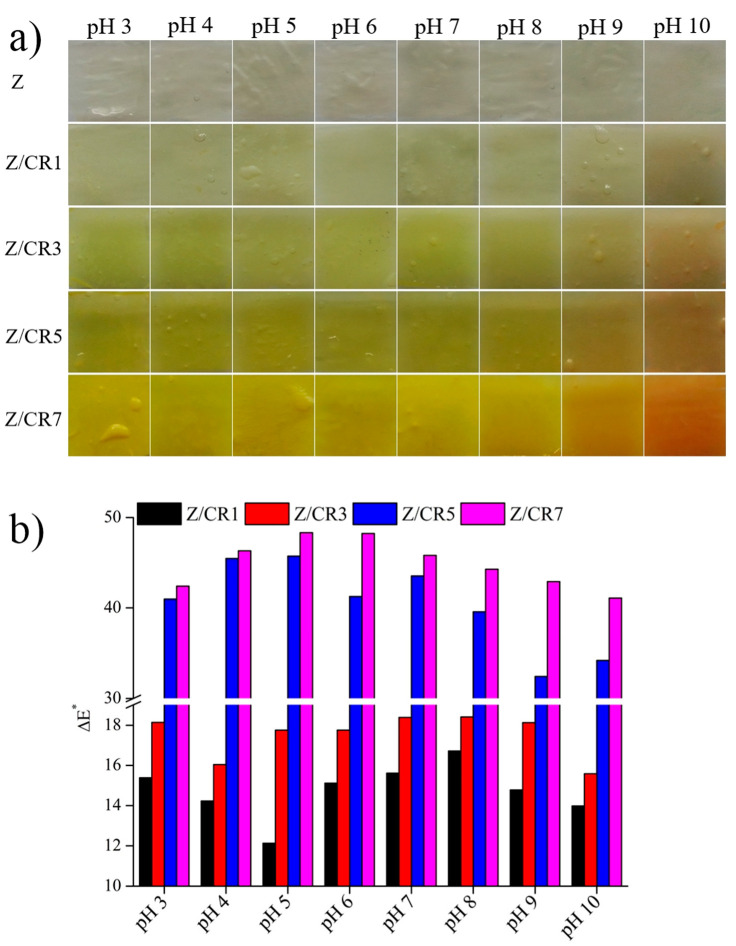
Appearance of zein films containing curcumin at different concentrations after immersion in different pH buffers (**a**) and color changes in the films (**b**).

The color response of films to pH variations is also important for their application as freshness indicators. To evaluate this property, films were immersed in buffer solutions ranging from pH 3 to 10. As illustrated in Figure 5a, pure zein films (Z) showed no visible change, whereas curcumin-enriched films exhibited progressive color variations, observable by the naked eye. The calculated ΔE* values confirmed that color differences increased with curcumin concentration and pH level (Figure 5b). The most pronounced color responses were observed in Z/CR5 and Z/CR7 films. This pH sensitivity arises from the structural transformation of curcumin, which exists predominantly in the keto form under acidic and neutral conditions but shifts to the enol form at alkaline pH [20]. Consequently, curcumin-based films appeared yellow at neutral or acidic pH and turned reddish-brown at alkaline values due to pH-dependent degradation of curcumin [9,26].

#### 3.1.5. Antioxidant Capacity

Antioxidant capacity is a crucial attribute of active packaging, as it helps to mitigate oxidative reactions responsible for rancidity, off-flavors, and discoloration in food products [24]. Curcumin, a natural polyphenolic compound, is widely recognized for its potent radical scavenging activity. Pure curcumin exhibits an exceptionally high antioxidant capacity of approximately 1046.66 µmol Trolox/g, making it a strong candidate for enhancing the oxidative stability of biopolymer films. In this study, the incorporation of curcumin into zein-based films significantly increased the antioxidant capacity in a concentration-dependent manner. Control films (Z) exhibited the lowest value (12.58 ± 0.22 µmol Trolox/g), while Z/CR7 films reached 24.18 ± 0.70 µmol Trolox/g, representing a marked improvement with increasing curcumin content. These results are consistent with previous findings, such as those by Tian et al. (2024) [27], who reported that antioxidant activity in zein/polysaccharide nanoparticle films was positively correlated with curcumin concentration. Similarly, Rachtanapun et al. (2021) [28] demonstrated enhanced antioxidant performance in chitosan-based films enriched with curcumin extract, further confirming the ability of curcumin to boost free radical scavenging capacity. The antioxidant efficiency of curcumin-loaded films is not solely determined by the concentration of curcumin but also depends on factors such as release dynamics, physicochemical interactions between curcumin and the polymer matrix, and microstructural organization [10]. The primary mechanism is linked to the hydrogen-donating ability of phenolic groups in curcumin, which neutralize free radicals [29]. Overall, zein–curcumin films exhibited significantly enhanced antioxidant properties compared to neat zein films, highlighting their potential as multifunctional active packaging materials capable of extending the shelf-life of perishable foods.

#### 3.1.6. Mechanical Properties

The mechanical properties, including tensile strength (TS), elongation at break (EAB), burst strength (BS), and burst deformation (BD), of zein-based films containing different concentrations of curcumin are summarized in Table 3. Pure zein films (Z) exhibited the highest TS value (12.62 MPa), which significantly decreased with increasing curcumin content, reaching the lowest value (6.76 MPa) in Z/CR7 films (*p* < 0.05). Conversely, the EAB values initially showed a slight increase up to 3% curcumin incorporation, followed by a marked increase at higher curcumin concentrations, indicating a pronounced plasticizing effect of curcumin. This opposite trend in TS and EAB can be attributed to the disruption of hydrogen bond cross-linking and weakening of intermolecular forces within the zein matrix, which enhanced the deformability of the films [27].

Zein films are known to have limited water resistance, and the increase in water uptake observed with curcumin addition likely contributed to plasticization, thereby enhancing flexibility while reducing brittleness and sudden crack propagation. In line with our findings, Wang, Xue, and Zhang (2019) [30] reported no significant changes in TS and EAB when 1% curcumin was incorporated into caseinate/zein nanocomposite films. By contrast, studies on chitosan/curcumin nanoparticle-based composite films demonstrated that both TS and EAB increased with curcumin addition [31], while Tian et al. (2024) [27] observed a significant reduction in TS coupled with an increase in EAB in zein/polysaccharide films at higher curcumin concentrations. Similarly, Ren et al. (2022) [32] showed that low curcumin concentrations improved TS and EAB in zein/chitosan/eugenol films, but further increases in curcumin reduced TS.

Other critical indicators of film resistance are puncture force (BS) and deformation (BD). In this study, both BS and BD values were lowest for the control films but significantly increased with curcumin incorporation (*p* < 0.05). The enhancement in BS and BD can be explained by the denser microstructure of curcumin-added films, which exhibited lower moisture content, resulting in tighter film formation and higher mechanical resilience. Furthermore, the plasticizing effect of curcumin, in conjunction with its contribution to higher water uptake, also favored greater BD values, consistent with the EAB trends. Previous work has shown that phenolic compounds incorporated into zein matrices enhance elongation and deformability by disrupting or weakening intermolecular hydrogen bonds, thereby increasing chain mobility within the polymer network [33].

#### 3.1.7. Thermal Properties

**Figure 6:**
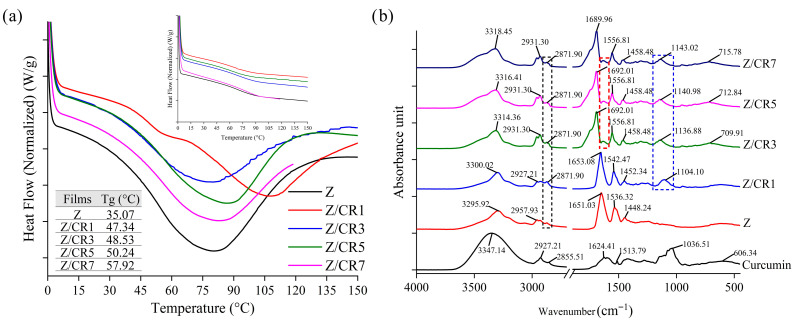
Thermal properties (**a**) and FTIR spectra (**b**) of zein films containing curcumin at different concentrations.

Zein, a corn-derived protein with favorable film-forming properties, inherently exhibits thermal stability. Upon incorporation of curcumin, significant changes in the thermal behavior of zein films were observed (Figure 6a). The initial thermal scan of neat zein films revealed a broad endothermic transition, typical of amorphous biopolymers, which is generally attributed to enthalpy molecular relaxation [34]. The glass transition temperature (Tg) of zein-based films was detected at 35.07 °C, and curcumin incorporation led to a progressive increase in Tg, reaching up to 57.92 °C in films containing 7% curcumin. This increase may be explained by the formation of hydrogen bonds and hydrophobic interactions between curcumin and zein, which promote tighter molecular packing and greater rigidity of the protein matrix, thereby increasing Tg [35].

Amorphous materials undergoing glass transition are thermodynamically unstable and tend to relax toward a lower energy state when maintained at temperatures just below their Tg, leading to the reduction in enthalpy or entropy. After reheating, the zein-based films displayed a disappearance of endothermic peaks, yielding relatively flat thermograms (Figure 6a).

The magnitude of the endothermic peaks was closely correlated with the moisture content of the films. The largest peak was observed in the curcumin-free zein film, which had the highest moisture content. In films with low moisture content, molecular mobility is restricted, resulting in broad endothermic relaxation peaks. Conversely, films with higher moisture content, particularly when exposed to temperatures close to Tg, exhibited increased chain mobility, producing sharper endothermic peaks rather than broad ones [34].

#### 3.1.8. FTIR Spectroscopy

The FTIR spectra of curcumin and curcumin-incorporated zein films were analyzed to elucidate molecular interactions between the polymer matrix and the active compound (Figure 6b). Pure curcumin exhibited strong absorption bands at 3347 cm^−1^ and 2927 cm^−1^, corresponding to phenolic O–H and –CH_2_ stretching, respectively. In its fingerprint region, characteristic peaks were observed at 1624 cm^−1^, 1513 cm^−1^, 1425 cm^−1^, 1280 cm^−1^, and 1036 cm^−1^, which were attributed to C=C and C=O conjugation of the aromatic ring, C=O stretching of the enol form, olefinic C–H in-plane bending of –CH_2_ bound to aromatic rings, enol C–O stretching of ether, and C–O–C stretching vibrations, respectively [35].

The pure zein films exhibited characteristic bands assigned to N–H stretching at 3295.92 cm^−1^, C–H stretching of aliphatic groups at 2957.93 and 2871.90 cm^−1^ (Amide-A and Amide-B), and peptide backbone vibrations at 1651.03, 1536.32, and 1448.24 cm^−1^, corresponding to amide-I (C=O stretching), amide-II (N–H bending), and amide-III (C–N stretching), respectively [36].

Upon the incorporation of curcumin, distinct changes were observed in both the intensities and positions of the peaks. New bands emerged, while shifts in Amide-A and Amide-B regions were recorded, indicating the formation of hydrogen bonds between curcumin’s phenolic –OH groups and the zein matrix. These blue shifts suggest a reduction in the strength of hydrogen bonding and an increase in bond vibration frequency, likely caused by partial substitution of zein–zein hydrogen bonds with weaker zein–curcumin or curcumin–curcumin interactions. At curcumin concentrations above 1%, shoulder peaks were detected in the Amide-A and Amide-I regions, further confirming molecular interactions. Additionally, a new band in the 1600–1550 cm^−1^ range appeared in films containing 3–7% curcumin, ascribable to C=C stretching of the aromatic benzene ring of curcumin. Peaks detected between 1104–1143 cm^−1^ were assigned to antisymmetric C–O–C stretching, while bands around 709–715 cm^−1^ were associated with methylene (–CH_2_) in-plane wobble vibrations.

These spectral changes demonstrate that curcumin was successfully integrated into the zein film structure through hydrogen bonding and hydrophobic interactions, reinforcing the compatibility between the active compound and the protein matrix.

#### 3.1.9. Scanning Electron Microscope (SEM)

**Figure 7:**
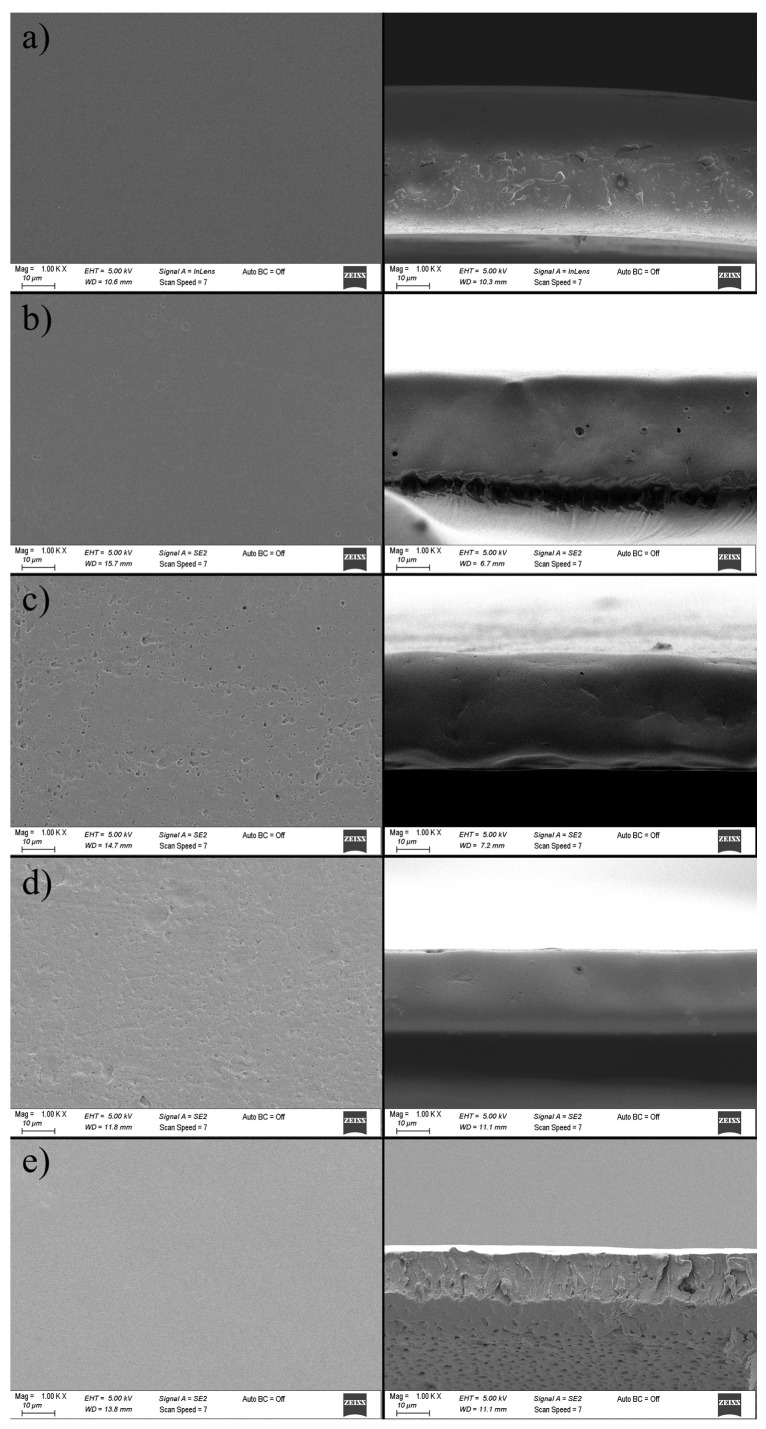
Effect of curcumin incorporation at different concentrations on surface (**left**) and cross-sectional (**right**) microstructure of zein-based films. (**a**) Z; (**b**) Z/CR1; (**c**) Z/CR3; (**d**) Z/CR5; (**e**) Z/CR7.

The mechanical, barrier, and optical properties of packaging films are closely linked to their microstructural composition, which is strongly influenced by drying conditions and the preparation method of the film-forming solutions. Films with rough, porous, and inhomogeneous microstructures generally exhibit weaker mechanical strength, higher opacity, and poorer gas barrier properties. Conversely, smooth, homogeneous, and compact microstructures result in improved mechanical strength, transparency, and enhanced barrier performance. The SEM micrographs of zein-based films, both at the surface and cross-sectional levels, are presented in Figure 7.

The neat zein film (without curcumin) displayed a smooth and uniform surface, free of pores. In contrast, the incorporation of curcumin up to 5% resulted in a rougher, more porous surface morphology, which can be attributed to solvent evaporation during drying, leading to the random aggregation and deposition of curcumin crystals within the film matrix [37]. Pores observed at the film surface were also evident in the cross-sectional images; however, pore propagation along the entire cross-section was not clearly discernible, likely due to polymer aggregation during solvent evaporation.

The cross-sectional micrographs revealed that the control zein film exhibited a relatively compact but rougher structure, whereas films containing up to 5% curcumin showed smoother and flatter morphologies. That could be explained by the plasticizing effect of curcumin, which enhanced film flexibility and water uptake, while hydrogen bonding interactions between zein and curcumin further contributed to microstructural modification.

Interestingly, although higher curcumin loading was expected to promote more heterogeneity, the 7% curcumin film exhibited a smoother and more homogeneous morphology. A similar phenomenon was reported for agar, chitosan, and carrageenan films enriched with curcumin, where curcumin did not significantly increase surface roughness [38]. Other studies showed that the curcumin addition in zein-based nanocomposite films induced more wrinkles and irregularities at the surface [27,30]. In the present work, however, no signs of curcumin aggregation were observed in either the surface or cross-sectional images, suggesting good miscibility and compatibility between curcumin and zein in the film-forming solution.

### 3.2. Application of Films for Monitoring Real-Time Freshness of Fish

#### 3.2.1. pH

The pH values of sea bream fillets during refrigerated storage are presented in Table 4. At day 0, the initial pH (6.29) was consistent with values reported in previous studies on fresh sea bream and other fish species [39,40]. The muscle pH of live fish is close to neutrality (~7.0), but postmortem values typically fall between 6.0 and 7.1 depending on species, season, and physiological state [41]. As expected, the pH of all treatments increased progressively during storage, reflecting microbial activity and the accumulation of volatile amines. The control group (C) exhibited the greatest increase, while fillets packaged with zein films, particularly Z/CR7, showed a slightly slower rise. Although differences between treatments on day 13 were not statistically significant, the overall trend confirmed that curcumin-enriched zein films moderated the pH increase compared to the untreated control. Similar gradual increases in pH during seafood spoilage have been widely reported [42,43]. For example, shrimp packaged with curcumin–anthocyanin colorimetric films reached a final pH of 7.73 after 10 days, compared to 6.29 initially [12].

#### 3.2.2. Thiobarbituric Acid Reactive Substances (TBARS)

TBARS values, reflecting the formation of malondialdehyde and other secondary lipid oxidation products, are also reported in Table 4. On day 0, sea bream fillets exhibited low TBARS values (0.121 mg MDA/kg), which increased during storage across all groups. The control group showed the highest accumulation, while films containing zein alone (Z) moderately slowed lipid oxidation, and the curcumin-enriched film (Z/CR7) showed the strongest inhibitory effect, with TBARS values less than half of the control by day 13. These results align with previous findings that active coatings or films enriched with antioxidants reduce oxidative rancidity in seafood [15,44]. Quality guidelines suggest that TBARS values below 5 mg MDA/kg indicate acceptable freshness [45]. In our study, all treatments remained well below this threshold, consistent with observations in other packaged sea bream systems [46]. It should also be noted that the TBARS assay lacks absolute specificity, as it can react with compounds beyond MDA. Nonetheless, the marked reduction observed in the Z/CR7 group demonstrates the effective antioxidant contribution of curcumin.

#### 3.2.3. Total Volatile Basic Nitrogen (TVB—N)

TVB—N is a widely used spoilage index in fish, reflecting the accumulation of volatile amines such as ammonia, trimethylamine, and dimethylamine through microbial and enzymatic degradation of proteins. As shown in Table 4, initial TVB—N values were typical of fresh sea bream fillets (~12 mg N/100 g). During storage, the control group (C) showed a sharp increase, surpassing 30 mg N/100 g—the generally accepted spoilage limit—by day 9 and exceeding 100 mg N/100 g by day 13. The neat zein film (Z) had a modest effect, delaying TVB—N accumulation by ~12% relative to control. In contrast, the curcumin-loaded film (Z/CR7) was significantly more effective, limiting TVB—N to ~85 mg N/100 g by day 13, representing a ~25% reduction compared with control. These findings indicate that Z/CR7 extended the shelf life by approximately three days. Comparable trends have been reported for active coatings, where chitosan nanofiber-coated sea bream fillets remained acceptable for 9 days, versus only 5 days in untreated controls [47]. Mechanistically, the superior performance of Z/CR7 can be attributed to the vapor-phase diffusion of curcumin phenolics into the headspace, where they exert antioxidant and antimicrobial effects. This slows microbial proliferation and deamination reactions responsible for volatile amine formation, whereas neat zein films provide only passive barrier protection.

#### 3.2.4. Optical Properties

Color change of packaging films during storage is a key parameter for monitoring food freshness. In this study, color analysis was performed on the films attached to the lids of fish containers (without direct contact with the fillets). The corresponding L*, a*, and b* values are shown in Table 5, while the visual appearance of the films throughout storage is illustrated in Figure 7.

The L* values of both film types decreased slightly during storage, indicating reduced brightness. All samples exhibited negative a* values, consistent with a greenish hue. However, the curcumin-containing films (Z/CR7) displayed significantly greater variations in a* and b* values compared with neat zein films, and these changes were clearly visible to the naked eye (*p* < 0.05). This finding confirms the superior sensitivity of curcumin as a natural pH indicator, in line with previous observations on starch-based curcumin films applied to fish fillets [10].

The observed changes are attributable to volatile nitrogenous compounds (ammonia, trimethylamine, dimethylamine) released during protein and lipid degradation. These compounds diffuse into the package headspace and dissolve in surface moisture of the indicator film, creating an alkaline environment that triggers colorimetric shifts [20,48]. Curcumin is highly responsive to such pH changes, as the deprotonation of its phenolic hydroxyl groups leads to the formation of phenoxide anions, resulting in visible color transitions [49]. In this study, the pH increase in sea bream from ~6.29 at day 0 to ~7.03 at day 13 (Table 4) correlated well with the progressive color change of Z/CR7 films.

Overall, these results demonstrate that curcumin-enriched zein films provide a reliable visual signal of freshness loss in fish, supporting their potential as intelligent colorimetric indicators for real-time spoilage monitoring [10,50].

#### 3.2.5. Microbial Analysis

Fresh fish is highly perishable due to its high-water activity, rich nutrient composition, nearly neutral pH, and the presence of autolytic enzymes. During refrigerated storage, spoilage is mainly driven by microbial proliferation, followed by enzymatic activity and, to a lesser extent, chemical processes such as lipid oxidation and hydrolysis [51].

At the beginning of storage, the total viable count (TVC), psychrotrophic count (PTC), and yeast and mold count (Y/M) were 5.40, 5.54, and 3.55 log CFU/g, respectively (Table 6). All microbial counts increased significantly with storage time. By day 13, both TVC and PTC values surpassed the generally accepted microbiological limit for fish products (6–7 log CFU/g), confirming the end of shelf life [10] (Chen et al., 2020). The control group (C) reached 6.48 log CFU/g (TVC) and 6.12 log CFU/g (PTC), while Z/CR7-treated samples showed very similar values (6.46 and 6.26 log CFU/g, respectively), indicating only marginal differences. Yeasts and molds also proliferated steadily, from an initial 3.55 log CFU/g to ~5.9 log CFU/g at day 13, with no significant inhibition by the films.

Although curcumin exhibits well-documented antimicrobial properties in vitro, its effectiveness when incorporated into zein-based films was limited under the conditions tested. The curcumin-enriched film (Z/CR7), attached to the inner surface of the package without direct contact with the fish, did not substantially delay microbial proliferation compared with control samples. This limited effect can be attributed to factors, such as curcumin concentration, diffusion kinetics, interactions with the food matrix, and the non-contact application mode [52].

Spoilage microorganisms not only increase the microbial load but also produce metabolites (e.g., ammonia, histamine, and cadaverine) that deteriorate sensory quality and lead to consumer rejection [43]. Protein breakdown by these microorganisms leads to the accumulation of total volatile basic nitrogen (TVB—N), which in turn, increases the pH of the fish tissue [26]. This accumulation of alkaline compounds—mainly ammonia and trimethylamine—creates the headspace conditions responsible for the color change observed in the curcumin films [41,48].

## 4. Conclusions

This study demonstrated that incorporating curcumin into zein-based edible films effectively enhanced their optical, antioxidant, and barrier properties while introducing pH sensitivity useful for intelligent food packaging. The improvements observed are consistent with previous evidence showing that phenolic compounds interact with protein matrices to modify structural and functional behavior. Among the formulations, the film containing 7% curcumin (Z/CR7) showed the best performance, providing a distinct colorimetric response that correlated with chemical indicators of sea bream spoilage. Although antimicrobial activity remained limited, the results confirm that curcumin-enriched zein films can function as natural, biodegradable freshness indicators. These findings highlight the potential of such bio-based composites to contribute to sustainable food quality monitoring and waste reduction in seafood packaging systems.

## Figures and Tables

**Table 1:** Thickness and Water vapor permeability (WVP).

Films	Thickness (mm)	MC (%)	SI (%)	Sol (%)	WVP (g·mm/m^2^·h·kPa)
Z	0.032 ± 0.001 ^b^	6.13 ± 0.22	26.87 ± 0.31 ^e^	20.18 ± 0.69 ^c^	0.110 ± 0.00 ^a^
Z/CR1	0.032 ± 0.004 ^b^	6.08 ± 0.52	49.28 ± 0.76 ^d^	23.36 ± 0.01 ^b^	0.106 ± 0.00 ^b^
Z/CR3	0.032 ± 0.002 ^b^	5.98 ± 0.49	56.33 ± 2.97 ^c^	23.40 ± 0.96 ^b^	0.096 ± 0.00 ^c^
Z/CR5	0.033 ± 0.001 ^ab^	5.71 ± 1.02	63.48 ± 2.15 ^b^	24.76 ± 0.91 ^ab^	0.093 ± 0.00 ^d^
Z/CR7	0.034 ± 0.004 ^a^	5.11 ± 0.42	76.28 ± 1.72 ^a^	24.99 ± 0.95 ^a^	0.085 ± 0.01 ^e^

Values are Mean ± Standard deviation. Different letters in the same column indicate significant differences in results (*p* < 0.05). Z: Zein film. Z/CR1: Zein film containing 1% curcumin. Z/CR3: Zein film containing 3% curcumin. Z/CR5: Zein film containing 5% curcumin. Z/CR7: Zein film containing 7% curcumin. MC: Moisture content; SI: Swelling index; Sol: Solubility; WVP: Water vapor permeability.

**Table 2:** Optical properties and antioxidant capacity of the films.

Films	L*	a*	b*	Opacity	AC (µmol Trolox/g)
Z	93.91 ± 0.19 ^b^	−5.75 ± 0.04 ^a^	27.17 ± 0.27 ^d^	2.35 ± 0.01 ^b^	12.58 ± 0.22 ^e^
Z/CR1	93.91 ± 0.06 ^b^	−10.48 ± 0.05 ^b^	40.38 ± 0.26 ^c^	2.27 ± 0.34 ^b^	14.07 ± 0.09 ^d^
Z/CR3	94.41 ± 0.11 ^a^	−15.57 ± 0.21 ^c^	56.70 ± 1.62 ^b^	3.27 ± 0.26 ^a^	19.01 ± 0.28 ^c^
Z/CR5	92.46 ± 0.56 ^d^	−16.52 ± 0.99 ^d^	89.61 ± 0.98 ^a^	3.29 ± 0.47 ^a^	22.05 ± 0.31 ^b^
Z/CR7	93.36 ± 0.19 ^c^	−17.90 ± 0.23 ^e^	90.51 ± 0.98 ^a^	4.01 ± 0.20 ^a^	24.18 ± 0.70 ^a^

Values are Mean ± Standard deviation. Different letters in the same column indicate significant differences in results (*p* < 0.05). AC: Antioxidant capacity; L*: Lightness; a*: Redness; b*: Yellowness.

**Table 3:** Mechanical properties of zein-based films containing curcumin.

Films	TS (MPa)	EAB (%)	BS (N)	BD (mm)
Z	12.62 ± 1.47 ^a^	10.25 ± 0.35 ^c^	1.54 ± 0.15 ^d^	1.64 ± 0.18 ^d^
Z/CR1	10.49 ± 1.01 ^a^	10.91 ± 0.87 ^c^	2.11 ± 0.17 ^c^	2.27 ± 0.12 ^c^
Z/CR3	9.53 ± 0.75 ^bc^	16.01 ± 1.65 ^c^	2.33 ± 0.19 ^b^	2.45 ± 0.12 ^bc^
Z/CR5	8.92 ± 0.26 ^c^	29.00 ± 4.80 ^b^	2.52 ± 0.21 ^ab^	2.72 ± 0.44 ^b^
Z/CR7	6.76 ± 0.38 ^d^	65.14 ± 8.50 ^a^	2.71 ± 0.26 ^a^	3.56 ± 0.28 ^a^

Values are Mean ± Standard Deviation. Different letters in the same column indicate significant differences (*p* < 0.05). TS: tensile strength; EAB: elongation at break; BS: burst strength; BD: burst deformation.

**Table 4:** Physicochemical parameters (pH, TBARS, TVB—N) and color attributes (L*, a*, b*) of sea bream fillets during refrigerated storage (4 °C).

Parameters	Treatments	Storage Time (Days)			
		0	3	8	13
pH	C	6.29 ± 0.05 ^C^	6.33 ± 0.04 ^C^	6.83 ± 0.19 ^B^	7.03 ± 0.12 ^A^
	Z	6.29 ± 0.05 ^C^	6.28 ± 0.02 ^C^	6.67 ± 0.13 ^B^	7.03 ± 0.11 ^A^
	Z/CR7	6.29 ± 0.05 ^C^	6.29 ± 0.04 ^C^	6.57 ± 0.16 ^B^	7.02 ± 0.13 ^A^
TBARS (mg MDA/g)	C	0.121 ± 0.01 ^B^	0.148 ± 0.03 ^B^	0.267 ± 0.04 ^aA^	0.253 ± 0.08 ^aA^
	Z	0.121 ± 0.01 ^C^	0.125 ± 0.02 ^C^	0.161 ± 0.01 ^bB^	0.196 ± 0.03 ^bA^
	Z/CR7	0.121 ± 0.01 ^B^	0.128 ± 0.02 ^B^	0.123 ± 0.01 ^bB^	0.146 ± 0.02 ^cA^
TVB—N (mg N/100 g)	C	11.92 ± 0.71 ^D^	20.87 ± 0.41 ^C^	47.78 ± 6.28 ^B^	113.84 ± 11.1 ^A^
	Z	11.92 ± 0.71 ^D^	18.37 ± 1.36 ^C^	52.77 ± 0.62 ^B^	107.15 ± 8.85 ^A^
	Z/CR7	11.92 ± 0.71 ^D^	19.72 ± 1.41 ^C^	44.69 ± 0.77 ^B^	84.12 ± 2.47 ^A^
L*	C	57.55 ± 0.66 ^B^	55.89 ± 1.51 ^BC^	55.66 ± 0.33 ^C^	59.87 ± 1.63 ^A^
	Z	57.55 ± 0.66	56.60 ± 2.59	58.96 ± 1.31	59.11 ± 2.47
	Z/CR7	57.55 ± 0.66	57.16 ± 0.43	57.50 ± 4.18	60.12 ± 0.31
a*	C	−2.54 ± 0.72 ^A^	−3.13 ± 0.06 ^cA^	−3.04 ± 0.12 ^aA^	−3.82 ± 0.01 ^aB^
	Z	−2.54 ± 0.72	−2.22 ± 0.09 ^a^	−2.49 ± 0.06 ^b^	−2.72 ± 0.02 ^b^
	Z/CR7	−2.54 ± 0.72 ^A^	−2.80 ± 0.02 b^A^	−3.71 ± 0.11 ^cB^	−4.55 ± 0.01 ^aC^
b*	C	4.58 ± 0.41 ^B^	4.96 ± 0.30 ^bB^	4.76 ± 0.47 ^cB^	9.48 ± 1.39 ^aA^
	Z	4.58 ± 0.41 ^C^	6.27 ± 0.94 ^aB^	7.32 ± 0.03 ^aA^	7.77 ± 0.03 ^bA^
	Z/CR7	4.58 ± 0.41	4.70 ± 0.52 ^b^	8.87 ± 0.55 ^b^	6.30 ± 0.27 ^c^

Values are mean ± standard deviation. Different lowercase superscripts within the same column indicate significant differences between treatments; different uppercase superscripts within the same row indicate significant differences over time (*p* < 0.05). L*: Lightness; a*: Redness; b*: Yellowness; C: control without film; Z: zein film; Z/CR7: zein film with 7% curcumin; TBARS: thiobarbituric acid reactive substances; TVB—N: total volatile basic nitrogen.

**Table 5:** Color parameters (L*, a*, b*) of zein films attached to container lids during refrigerated storage (4 °C).

Films		Storage Period (Days)			
		0	3	8	13
Z	*L*	95.27 ± 0.03 ^a^	94.99 ± 0.06 ^a^	92.28 ± 0.47 ^d^	93.29 ± 0.51 ^c^
	a*	−1.04 ± 0.03 ^a^	−1.64 ± 0.12 ^c^	−1.32 ± 0.04 ^b^	−1.30 ± 0.02 ^b^
	b*	7.65 ± 0.08 ^c^	11.82 ± 0.7 ^ab^	12.14 ± 0.03 a	11.57 ± 0.18 ^b^
Z/CR7	*L*	92.69 ± 0.01 ^a^	91.98 ± 0.29 ^b^	91.17 ± 0.31 ^c^	90.84 ± 0.09 ^c^
	a*	−13.36 ± 0.01 ^c^	−10.61 ± 1.18 ^b^	−11.02 ± 0.12 ^b^	−10.16 ± 0.07 ^b^
	b*	74.42 ± 0.03 ^c^	72.95 ± 0.27 ^d^	75.93 ± 0.54 ^b^	77.75 ± 0.05 ^a^

Values are mean ± standard deviation. Different letters within the same column indicate significant differences (*p* < 0.05). L*: Lightness; a*: Redness; b*: Yellowness.

**Table 6:** Microbiological parameters of sea bream fillets during refrigerated storage (4 °C).

Parameters	Treatments	Storage Time (Days)			
		0	3	8	13
TVC (CFU/g)	C	5.40 ± 0.02 ^C^	5.41 ± 0.01 ^bC^	6.34 ± 0.09 ^B^	6.48 ± 0.03 ^A^
	Z	5.40 ± 0.02 ^B^	6.22 ± 0.21 ^aA^	6.39 ± 0.03 ^A^	6.44 ± 0.06 ^A^
	Z/CR7	5.40 ± 0.02 ^C^	6.33 ± 0.1 ^aB^	6.40 ± 0.13 ^AB^	6.46 ± 0.03 ^A^
PTC (CFU/g)	C	5.54 ± 0.05 ^B^	5.69 ± 0.11 ^aB^	6.23 ± 0.03 ^aA^	6.12 ± 0.13 ^bA^
	Z	5.54 ± 0.05 ^B^	5.39 ± 0.11 ^bC^	6.26 ± 0.01 ^aA^	6.30 ± 0.02 ^aA^
	Z/CR7	5.54 ± 0.05 ^D^	5.28 ± 0.07 ^bC^	6.14 ± 0.03 ^bB^	6.26 ± 0.03 ^abA^
Y/M (CFU/g)	C	3.55 ± 0.05 ^C^	5.30 ± 0.02 ^aB^	5.36 ± 0.02 ^aB^	5.74 ± 0.11 ^A^
	Z	3.55 ± 0.05 ^D^	4.55 ± 0.04 ^bC^	5.22 ± 0.04 ^bB^	5.88 ± 0.08 ^A^
	Z/CR7	3.55 ± 0.05 ^D^	4.49 ± 0.26 ^bC^	5.41 ± 0.04 ^aB^	5.87 ± 0.03 ^A^

Values are mean ± standard deviation. Different lowercase superscripts within a column indicate significant differences between treatments at the same storage time (*p* < 0.05), while different uppercase superscripts within a row indicate significant differences among storage times for the same treatment (*p* < 0.05). TVC: total viable count; PTC: psychrotrophic count; Y/M: yeast and mold count.

## Data Availability

The original contributions presented in the study are included in the article; further inquiries can be directed to the corresponding authors.

## References

[1] Ge X., Huang X., Zhou L., Wang Y. (2022). Essential oil-loaded antimicrobial and antioxidant zein/poly(lactic acid) film as active food packaging. Food Packag. Shelf Life.

[2] Kaur J., Singh J., Rasane P., Gupta P., Kaur S., Sharma N., Sowdhanya D. (2023). Natural additives as active components in edible films and coatings. Food Biosci..

[3] Etxabide A., Kilmartin P.A., Maté J.I. (2021). Color stability and pH-indicator ability of curcumin, anthocyanin and betanin containing colorants under different storage conditions for intelligent packaging development. Food Control.

[4] Wei L., Dou M., Zhang W., Xu X., Chen H., Zhang Z. (2024). Characterization of zein-based films plasticized with deep eutectic solvents and their use in the preservation of harvested mango fruit. Food Hydrocoll..

[5] Yilmaz M.T., Kul E., Saricaoglu F.T., Odabas H.I., Taylan O., Dertli E. (2024). Deep eutectic solvent as plasticizing agent for the zein based films. Food Packag. Shelf Life.

[6] Lan X., Zhang X., Wang L., Wang H., Hu Z., Ju X., Yuan Y. (2023). A review of food preservation based on zein: The perspective from application types of coating and film. Food Chem..

[7] Urošević M., Nikolić L., Gajić I., Nikolić V., Dinić A., Miljković V. (2022). Curcumin: Biological activities and modern pharmaceutical forms. Antibiotics.

[8] Zhang L., Chen D., Yu D., Regenstein J.M., Jiang Q., Dong J., Chen W., Xia W. (2022). Modulating physicochemical, antimicrobial and release properties of chitosan/zein bilayer films with curcumin/nisin-loaded pectin nanoparticles. Food Hydrocoll..

[9] Zheng D., Huang C., Huang H., Zhao Y., Khan M.R.U., Zhao H., Huang L. (2020). Antibacterial mechanism of curcumin: A review. Chem. Biodivers..

[10] Chen H.-z., Zhang M., Bhandari B., Yang C.-H. (2020). Novel pH-sensitive films containing curcumin and anthocyanins to monitor fish freshness. Food Hydrocoll..

[11] Ezati P., Rhim J.-W. (2020). pH-responsive pectin-based multifunctional films incorporated with curcumin and sulfur nanoparticles. Carbohydr. Polym..

[12] Mohseni-Shahri F.S., Moeinpour F. (2023). Development of a pH-sensing indicator for shrimp freshness monitoring: Curcumin and anthocyanin-loaded gelatin films. Food Sci. Nutr..

[13] Passi S., Ricci R., Cataudella S., Ferrante I., De Simone F., Rastrelli L. (2004). Fatty acid pattern, oxidation product development, and antioxidant loss in muscle tissue of rainbow trout and *Dicentrarchus labrax* during growth. J. Agric. Food Chem..

[14] Rico D., Albertos I., Martinez-Alvarez O., Lopez-Caballero M.E., Martin-Diana A.B. (2020). Use of Sea Fennel as a Natural Ingredient of Edible Films for Extending the Shelf Life of Fresh Fish Burgers. Molecules.

[15] Tural S., Turhan S. (2017). Effect of anchovy by-product protein coating incorporated with thyme essential oil on the shelf life of anchovy (*Engraulis encrasicolus* L.) fillets. Food Sci. Biotechnol..

[16] (2003). Standard Test Method for Water Vapor Transmission of Materials.

[17] Kurt A., Kahyaoglu T. (2014). Characterization of a new biodegradable edible film made from salep glucomannan. Carbohydr. Polym..

[18] (2001). Standard Test Method for Tensile Properties of Thin Plastic Sheeting.

[19] Zhang J., Zou X., Zhai X., Huang X., Jiang C., Holmes M. (2019). Preparation of an intelligent pH film based on biodegradable polymers and roselle anthocyanins for monitoring pork freshness. Food Chem..

[20] Guo C., Li Y., Zhang H., Zhang Q., Wu X., Wang Y., Sun F., Shi S., Xia X. (2024). A review on improving the sensitivity and color stability of naturally sourced pH-sensitive indicator films. Compr. Rev. Food Sci. Food Saf..

[21] Sasaki H., Sunagawa Y., Takahashi K., Imaizumi A., Fukuda H., Hashimoto T., Wada H., Katanasaka Y., Kakeya H., Fujita M. (2011). Innovative preparation of curcumin for improved oral bioavailability. Biol. Pharm. Bull..

[22] Moradi M., Tajik H., Razavi Rohani S.M., Mahmoudian A. (2016). Antioxidant and antimicrobial effects of zein edible film impregnated with *Zataria multiflora* Boiss. essential oil and monolaurin. LWT—Food Sci. Technol..

[23] Esatbeyoglu T., Huebbe P., Ernst I.M., Chin D., Wagner A.E., Rimbach G. (2012). Curcumin—From molecule to biological function. Angew. Chem. Int. Ed. Engl..

[24] Wang R., Chen Z., Shu Y., Wang Y., Wang W., Zhu H., Su J., Ma Q. (2024). Apple pectin-based active films to preserve oil: Effects of naturally branched phytoglycogen-curcumin host. Int. J. Biol. Macromol..

[25] Said N.S., Lee W.Y. (2025). Pectin-Based Active and Smart Film Packaging: A Comprehensive Review of Recent Advancements in Antimicrobial, Antioxidant, and Smart Colorimetric Systems for Enhanced Food Preservation. Molecules.

[26] Liu D., Dang S., Zhang L., Munsop K., Li X. (2021). Corn starch/polyvinyl alcohol based films incorporated with curcumin-loaded Pickering emulsion for application in intelligent packaging. Int. J. Biol. Macromol..

[27] Tian Y., Yang X., Cao C., Lv Z., Han C., Guo Q., Duan Y., Zhang J. (2024). Improved antioxidant activities of edible films by curcumin-containing with zein/polysaccharide. Food Biosci..

[28] Rachtanapun P., Klunklin W., Jantrawut P., Jantanasakulwong K., Phimolsiripol Y., Seesuriyachan P., Leksawasdi N., Chaiyaso T., Ruksiriwanich W., Phongthai S. (2021). Characterization of Chitosan Film Incorporated with Curcumin Extract. Polymers.

[29] Zia J., Paul U.C., Heredia-Guerrero J.A., Athanassiou A., Fragouli D. (2019). Low-density polyethylene/curcumin melt extruded composites with enhanced water vapor barrier and antioxidant properties for active food packaging. Polymer.

[30] Wang L., Xue J., Zhang Y. (2019). Preparation and characterization of curcumin loaded caseinate/zein nanocomposite film using pH-driven method. Ind. Crops Prod..

[31] Xin S., Xiao L., Dong X., Li X., Wang Y., Hu X., Sameen D.E., Qin W., Zhu B. (2020). Preparation of chitosan/curcumin nanoparticles based zein and potato starch composite films for Schizothorax prenati fillet preservation. Int. J. Biol. Macromol..

[32] Ren M., Cai Z., Chen L., Wahia H., Zhang L., Wang Y., Yu X., Zhou C. (2022). Preparation of zein/chitosan/eugenol/curcumin active films for blueberry preservation. Int. J. Biol. Macromol..

[33] Arcan I., Yemenicioğlu A. (2011). Incorporating phenolic compounds opens a new perspective to use zein films as flexible bioactive packaging materials. Food Res. Int..

[34] Mouzakitis C.-K., Sereti V., Matsakidou A., Kotsiou K., Biliaderis C.G., Lazaridou A. (2022). Physicochemical properties of zein-based edible films and coatings for extending wheat bread shelf life. Food Hydrocoll..

[35] Monkos K. (2015). On the Possibility of Indirect Determination of the Glass Transition Temperature of Proteins from Viscosity Measurements and Avramov’s Model. Curr. Top. Biophys..

[36] Wang H., Hao L., Wang P., Chen M., Jiang S., Jiang S. (2017). Release kinetics and antibacterial activity of curcumin loaded zein fibers. Food Hydrocoll..

[37] Liu J., Wang H., Wang P., Guo M., Jiang S., Li X., Jiang S. (2018). Films based on κ-carrageenan incorporated with curcumin for freshness monitoring. Food Hydrocoll..

[38] Roy S., Rhim J.-W. (2020). Preparation of carbohydrate-based functional composite films incorporated with curcumin. Food Hydrocoll..

[39] Duran-Montgé P., Permanyer M., Belletti N. (2015). Refrigerated or Superchilled Skin-Packed Sea Bream (*Sparus aurata*) Compared with Traditional Unpacked Storage on Ice with Regard to Physicochemical, Microbial and Sensory Attributes. J. Food Process. Preserv..

[40] Ünal Şengör G.F., Balaban M.O., Ceylan Z., Doğruyol H. (2018). Determination of shelf life of gilthead seabream (*Sparus aurata*) with time temperature indicators. J. Food Process. Preserv..

[41] Kılınc B., Caklı S., Cadun A., Dıncer T., Tolasa S. (2007). Comparison of effects of slurry ice and flake ice pretreatments on the quality of aquacultured sea bream (*Sparus aurata*) and sea bass (*Dicentrarchus labrax*) stored at 4 °C. Food Chem..

[42] Kakaei S., Shahbazi Y. (2016). Effect of chitosan-gelatin film incorporated with ethanolic red grape seed extract and Ziziphora clinopodioides essential oil on survival of Listeria monocytogenes and chemical, microbial and sensory properties of minced trout fillet. LWT—Food Sci. Technol..

[43] Sobhan A., Muthukumarappan K., Wei L. (2022). A biopolymer-based pH indicator film for visually monitoring beef and fish spoilage. Food Biosci..

[44] Raeisi M., Tajik H., Aliakbarlu J., Mirhosseini S.H., Hosseini S.M.H. (2015). Effect of carboxymethyl cellulose-based coatings incorporated with Zataria multiflora Boiss. essential oil and grape seed extract on the shelf life of rainbow trout fillets. LWT—Food Sci. Technol..

[45] Sallam K.I. (2007). Antimicrobial and antioxidant effects of sodium acetate, sodium lactate, and sodium citrate in refrigerated sliced salmon. Food Control.

[46] Iacumin L., Jayasinghe A.S., Pellegrini M., Comi G. (2022). Evaluation of different techniques, including modified atmosphere, under vacuum packaging, washing, and *Latilactobacillus sakei* as a Bioprotective Agent, to increase the shelf-life of fresh gutted sea bass (*Dicentrarchus labrax*) and sea bream (*Sparus aurata*) stored at 6 ± 2 °C. Biology.

[47] Ceylan Z., Sengor G.F.U., Yilmaz M.T. (2017). A Novel Approach to Limit Chemical Deterioration of Gilthead Sea Bream (Sparus aurata) Fillets: Coating with Electrospun Nanofibers as Characterized by Molecular, Thermal, and Microstructural Properties. J. Food Sci..

[48] Zhou X., Yu X., Xie F., Fan Y., Xu X., Qi J., Xiong G., Gao X., Zhang F. (2021). pH-responsive double-layer indicator films based on konjac glucomannan/camellia oil and carrageenan/anthocyanin/curcumin for monitoring meat freshness. Food Hydrocoll..

[49] Tang T., Zhang M., Mujumdar A.S., Li C. (2024). 3D printed curcumin-based composite film for monitoring fish freshness. Food Packag. Shelf Life.

[50] Li H., Zhang X., Tan S., Tan G., Zhang H., Xia N., Jiang L., Ren H., Rayan A.M. (2022). Intelligent colorimetric soy protein isolate-based films incorporated with curcumin through an organic solvent-free pH-driven method: Properties, molecular interactions, and application. Food Hydrocoll..

[51] Socaciu M.-I., Semeniuc C.A., Vodnar D.C. (2018). Edible films and coatings for fresh fish packaging: Focus on quality changes and shelf-life extension. Coatings.

[52] Tkaczewska J., Kulawik P., Nowak N., Grzebieniarz W., Krzyściak P., Tadele W., Tadesse E.E., Szram R., Guzik P., Jamróz E. (2024). Comparing the effects of duo-functional triple-layer films enriched with different sources of curcumin on the shelf-life of fish. Foods.

